# Supplementary material for: Annual replication is essential in evaluating the response of the soil microbiome to the genetic modification of maize in different biogeographical regions
Source: PLoS One. 2019 Dec 17;14(12):e0222737. doi: 10.1371/journal.pone.0222737 (PMC6917299; doi:10.1371/journal.pone.0222737)
Supplement: S1 Table — (DOCX) [file pone.0222737.s002.docx]

**Supporting information**

S1 Table Sequences of primers and probes used in qPCR

|  | Primer/Probe | Reference |
| --- | --- | --- |
| Bacterial 16S rRNA  468 bp | BAC338F (5’-ACTCCTACGGGAGGCAG-3’)  BAC516F (5’-TGCCAGCAGCCGCGGTAATAC-3’)  BAC805R (5’-GACTACCAGGGTATCTAATCC-3’) | Yu et al., 2005 |
| Archaeal 16S rRNA  273 bp | ARC787F (5’- ATTAGATACCCSBGTAGTCC-3’)  ARC915F (5’- AGGAATTGGCGGGGGAGCAC-3’)  ARC1059R (5’-GCCATGCACCWCCTCT-3’) | Yu et al., 2005 |
| Fungal ITS  300-500 bp | NSI1 (5’- GATTGAATGGCTTAGTGAGG-3’)  58A2R (5’- CTGCGTTCTTCATCGAT-3’) | Martin & Rygiewicz, 2005 |
| *nirS*  426 bp | nirSCd3a-F (5’- AACGYSAAGGARACSGG-3’)  nirSR3cd-R (5’- GASTTCGGRTGSGTCTTSAYGAA -3’) | Kandeler et al., 2006 |
| *nirK*  165 bp | nirK876-F (5’- ATYGGCGGMVAYGGCGA -3’)  nirK1040-R (5’- GCCTCGATCAGRTTRTGGTT -3’) | Henry et al., 2004 |
