# Supplementary material for: Annual replication is essential in evaluating the response of the soil microbiome to the genetic modification of maize in different biogeographical regions
Source: PLoS One. 2019 Dec 17;14(12):e0222737. doi: 10.1371/journal.pone.0222737 (PMC6917299; doi:10.1371/journal.pone.0222737)
Supplement: S2 Table — Relative abundances are indicated as percentages (average ± standard deviation). The p-values are from Welch’s t-tests from ALDEx2 with Benjamini-Hochberg correction. The IDs of the nearest matching sequences from the FunGene nirK reference database and their source organisms are included. (DOCX) [file pone.0222737.s003.docx]

**Supporting information**

S2 Table The *nirK* translated sequence variants (TSVs) differentially abundant between the non-BT and BT samples from the Slovakian site in 2014.

**A. TSVs with higher abundance in the non-BT samples**

| TSV# | Reference ID | Source organism | Relative abundance | | Fold change | *p* |
| --- | --- | --- | --- | --- | --- | --- |
|  |  |  | non-BT | BT |  |  |
| TSV6 | Q01537 | Neorhizobium galegae | 4.399 ± 0.378 | 0.009 ± 0.023 | 493.5 | 0.0001 |
| TSV2 | YP_004614534 | Mesorhizobium opportunistum | 2.97 ± 0.499 | 0.476 ± 0.273 | 6.2 | 0.0007 |
| TSV3 | ACM24772 | Sinorhizobium sp. NP1 | 2.844 ± 0.428 | 0.009 ± 0.023 | 314.3 | 0.0001 |
| TSV243 | YP_001242619 | Bradyrhizobium sp. BTAi1 | 1.645 ± 0.276 | 0.002 ± 0.008 | 663.9 | 0.0001 |
| TSV15 | YP_001314809 | Sinorhizobium medicae WSM419 | 1.484 ± 0.262 | 0.005 ± 0.016 | 299.4 | 0.0002 |
| TSV21 | YP_001314809 | Sinorhizobium medicae WSM419 | 1.07 ± 0.101 | not detected | - | 0.0002 |
| TSV18 | YP_002825538 | Sinorhizobium fredii NGR234 | 0.946 ± 0.159 | 0.194 ± 0.379 | 4.9 | 0.0078 |
| TSV41 | Q01537 | Neorhizobium galegae | 0.862 ± 0.143 | not detected | - | 0.0002 |
| TSV342 | ACF98123 | uncultured bacterium 1062 | 0.73 ± 0.196 | not detected | - | 0.0002 |
| TSV31 | ZP_03523451 | Rhizobium etli GR56 | 0.687 ± 0.104 | 0.004 ± 0.009 | 173.6 | 0.0004 |
| TSV122 | YP_004134222 | Mesorhizobium ciceri biovar biserrulae WSM1271 | 0.62 ± 0.089 | 0.007 ± 0.012 | 91.0 | 0.0005 |
| TSV48 | YP_002825538 | Sinorhizobium fredii NGR234 | 0.599 ± 0.078 | 0.007 ± 0.017 | 81.6 | 0.0005 |
| TSV542 | ACF98152 | uncultured bacterium 1116 | 0.543 ± 0.154 | 0.002 ± 0.008 | 219.1 | 0.0005 |
| TSV47 | YP_001314809 | Sinorhizobium medicae WSM419 | 0.423 ± 0.096 | not detected | - | 0.0005 |
| TSV169 | YP_001242619 | Bradyrhizobium sp. BTAi1 | 0.377 ± 0.104 | 0.008 ± 0.013 | 49.5 | 0.0013 |
| TSV207 | 1NPJ_A | Alcaligenes Faecalis | 0.376 ± 0.071 | not detected | - | 0.0006 |
| TSV59 | YP_001314809 | Sinorhizobium medicae WSM419 | 0.372 ± 0.108 | not detected | - | 0.0005 |
| TSV729 | ACF98152 | uncultured bacterium 1116 | 0.349 ± 0.055 | not detected | - | 0.0006 |
| TSV579 | YP_001242619 | Bradyrhizobium sp. BTAi1 | 0.334 ± 0.096 | 0.006 ± 0.01 | 55.9 | 0.0017 |
| TSV65 | YP_004614534 | Mesorhizobium opportunistum | 0.321 ± 0.077 | 0.052 ± 0.088 | 6.1 | 0.0132 |
| TSV81 | NP_435927 | Sinorhizobium meliloti 1021 | 0.32 ± 0.035 | not detected | - | 0.0008 |
| TSV60 | ACM24772 | Sinorhizobium sp. NP1 | 0.307 ± 0.064 | not detected | - | 0.0009 |
| TSV385 | ZP_03523451 | Rhizobium etli GR56 | 0.305 ± 0.12 | 0.002 ± 0.008 | 123.0 | 0.0014 |
| TSV824 | ACF98123 | uncultured bacterium 1062 | 0.275 ± 0.097 | not detected | - | 0.0011 |
| TSV116 | ACF98123 | uncultured bacterium 1062 | 0.267 ± 0.041 | 0.002 ± 0.008 | 107.6 | 0.0012 |
| TSV876 | YP_004614534 | Mesorhizobium opportunistum | 0.242 ± 0.092 | not detected | - | 0.0012 |
| TSV76 | YP_001314809 | Sinorhizobium medicae WSM419 | 0.239 ± 0.067 | not detected | - | 0.0014 |
| TSV963 | ACF98152 | uncultured bacterium 1116 | 0.22 ± 0.067 | 0.005 ± 0.016 | 44.3 | 0.0025 |
| TSV80 | YP_004134222 | Mesorhizobium ciceri biovar biserrulae WSM1271 | 0.219 ± 0.086 | not detected | - | 0.0017 |
| TSV527 | ACF98123 | uncultured bacterium 1062 | 0.214 ± 0.069 | not detected | - | 0.0015 |
| TSV232 | YP_004134222 | Mesorhizobium ciceri biovar biserrulae WSM1271 | 0.203 ± 0.066 | 0.002 ± 0.006 | 111.6 | 0.0023 |
| TSV100 | YP_001314809 | Sinorhizobium medicae WSM419 | 0.193 ± 0.063 | not detected | - | 0.0018 |
| TSV149 | ACF98123 | uncultured bacterium 1062 | 0.191 ± 0.053 | 0.04 ± 0.076 | 4.8 | 0.0226 |
| TSV159 | ACF98123 | uncultured bacterium 1062 | 0.19 ± 0.048 | not detected | - | 0.0018 |
| TSV85 | ACF98123 | uncultured bacterium 1062 | 0.178 ± 0.06 | 0.004 ± 0.009 | 43.6 | 0.0030 |
| TSV968 | YP_004134222 | Mesorhizobium ciceri biovar biserrulae WSM1271 | 0.176 ± 0.062 | not detected | - | 0.0021 |
| TSV311 | ACF98152 | uncultured bacterium 1116 | 0.165 ± 0.038 | 0.003 ± 0.009 | 55.7 | 0.0038 |
| TSV110 | YP_674799 | Chelativorans sp. BNC1 | 0.151 ± 0.071 | not detected | - | 0.0029 |
| TSV153 | Q01537 | Neorhizobium galegae | 0.15 ± 0.073 | not detected | - | 0.0031 |
| TSV79 | NP_435927 | Sinorhizobium meliloti 1021 | 0.15 ± 0.045 | not detected | - | 0.0030 |
| TSV108 | ACF98123 | uncultured bacterium 1062 | 0.149 ± 0.051 | 0.009 ± 0.012 | 15.7 | 0.0098 |
| TSV765 | YP_004134222 | Mesorhizobium ciceri biovar biserrulae WSM1271 | 0.147 ± 0.055 | not detected | - | 0.0033 |
| TSV91 | Q01537 | Neorhizobium galegae | 0.146 ± 0.047 | not detected | - | 0.0032 |
| TSV94 | ACF98123 | uncultured bacterium 1062 | 0.144 ± 0.045 | not detected | - | 0.0032 |
| TSV7 | YP_004134222 | Mesorhizobium ciceri biovar biserrulae WSM1271 | 0.142 ± 0.042 | 0.011 ± 0.02 | 12.6 | 0.0147 |
| TSV78 | ACF98123 | uncultured bacterium 1062 | 0.138 ± 0.042 | not detected | - | 0.0032 |
| TSV505 | ACF98152 | uncultured bacterium 1116 | 0.132 ± 0.068 | 0.002 ± 0.008 | 53.1 | 0.0060 |
| TSV1254 | ACF98123 | uncultured bacterium 1062 | 0.131 ± 0.056 | 0.003 ± 0.009 | 46.9 | 0.0058 |
| TSV133 | YP_004614534 | Mesorhizobium opportunistum | 0.13 ± 0.047 | 0.01 ± 0.024 | 13.2 | 0.0120 |
| TSV1284 | YP_004110634 | Rhodopseudomonas palustris | 0.126 ± 0.052 | not detected | - | 0.0042 |
| TSV118 | YP_004614534 | Mesorhizobium opportunistum | 0.124 ± 0.067 | 0.007 ± 0.024 | 16.7 | 0.0103 |
| TSV148 | YP_002825538 | Sinorhizobium fredii NGR234 | 0.121 ± 0.041 | not detected | - | 0.0049 |
| TSV267 | ACF98123 | uncultured bacterium 1062 | 0.12 ± 0.049 | not detected | - | 0.0043 |
| TSV75 | YP_004614534 | Mesorhizobium opportunistum | 0.12 ± 0.033 | not detected | - | 0.0044 |
| TSV1337 | YP_665890 | Chelativorans sp. BNC1 | 0.118 ± 0.051 | not detected | - | 0.0048 |
| TSV210 | YP_004614534 | Mesorhizobium opportunistum | 0.117 ± 0.063 | not detected | - | 0.0088 |
| TSV184 | ACF98088 | uncultured bacterium 1042 | 0.114 ± 0.026 | not detected | - | 0.0051 |
| TSV142 | YP_004614534 | Mesorhizobium opportunistum | 0.113 ± 0.04 | 0.007 ± 0.01 | 15.7 | 0.0181 |
| TSV228 | ACM24772 | Sinorhizobium sp. NP1 | 0.111 ± 0.049 | not detected | - | 0.0051 |
| TSV188 | Q01537 | Neorhizobium galegae | 0.11 ± 0.047 | not detected | - | 0.0059 |
| TSV940 | ACF98123 | uncultured bacterium 1062 | 0.105 ± 0.03 | 0.005 ± 0.015 | 21.8 | 0.0104 |
| TSV157 | YP_001314809 | Sinorhizobium medicae WSM419 | 0.105 ± 0.033 | not detected | - | 0.0068 |
| TSV187 | YP_004614534 | Mesorhizobium opportunistum | 0.104 ± 0.046 | not detected | - | 0.0065 |
| TSV176 | YP_002825538 | Sinorhizobium fredii NGR234 | 0.101 ± 0.031 | not detected | - | 0.0071 |
| TSV1423 | YP_004614534 | Mesorhizobium opportunistum | 0.1 ± 0.024 | not detected | - | 0.0057 |
| TSV214 | ACM24772 | Sinorhizobium sp. NP1 | 0.091 ± 0.056 | not detected | - | 0.0140 |
| TSV257 | ACF98123 | uncultured bacterium 1062 | 0.085 ± 0.034 | not detected | - | 0.0122 |
| TSV312 | NP_435927 | Sinorhizobium meliloti 1021 | 0.084 ± 0.024 | not detected | - | 0.0088 |
| TSV304 | ACF98123 | uncultured bacterium 1062 | 0.083 ± 0.032 | not detected | - | 0.0107 |
| TSV436 | YP_665890 | Chelativorans sp. BNC1 | 0.082 ± 0.036 | not detected | - | 0.0117 |
| TSV1162 | NP_949481 | Rhodopseudomonas palustris | 0.082 ± 0.032 | not detected | - | 0.0125 |
| TSV791 | YP_473141 | Rhizobium etli | 0.079 ± 0.039 | 0.002 ± 0.008 | 32.0 | 0.0216 |
| TSV1659 | YP_004134222 | Mesorhizobium ciceri biovar biserrulae WSM1271 | 0.075 ± 0.017 | not detected | - | 0.0115 |
| TSV246 | ACF98123 | uncultured bacterium 1062 | 0.073 ± 0.016 | not detected | - | 0.0135 |
| TSV162 | ACM24772 | Sinorhizobium sp. NP1 | 0.073 ± 0.044 | not detected | - | 0.0181 |
| TSV294 | YP_002825538 | Sinorhizobium fredii NGR234 | 0.073 ± 0.036 | not detected | - | 0.0175 |
| TSV836 | NP_949481 | Rhodopseudomonas palustris | 0.072 ± 0.029 | not detected | - | 0.0196 |
| TSV1572 | ACF98123 | uncultured bacterium 1062 | 0.071 ± 0.026 | not detected | - | 0.0164 |
| TSV1650 | YP_004614534 | Mesorhizobium opportunistum | 0.07 ± 0.032 | not detected | - | 0.0178 |
| TSV325 | ACM24772 | Sinorhizobium sp. NP1 | 0.067 ± 0.031 | not detected | - | 0.0228 |

**B: TSVs with higher abundance in the BT samples**

| TSV# | Reference ID | Source organism | Relative abundance | | Fold change | *p* |
| --- | --- | --- | --- | --- | --- | --- |
|  |  |  | non-BT | BT |  |  |
| TSV17 | ACF98152 | uncultured bacterium 1116 | 1.723 ± 0.681 | 8.212 ± 7.027 | 4.8 | 0.0005 |
| TSV27 | YP_004134222 | Mesorhizobium ciceri biovar biserrulae WSM1271 | 2.209 ± 0.863 | 5.379 ± 1.430 | 2.4 | 1.87E-06 |
| TSV33 | YP_004134222 | Mesorhizobium ciceri biovar biserrulae WSM1271 | 2.057 ± 0.674 | 4.712 ± 1.639 | 2.3 | 2.45E-07 |
| TSV4 | NP_949481 | Rhodopseudomonas palustris | 2.317 ± 0.503 | 4.233 ± 1.534 | 1.8 | 3.47E-06 |
| TSV51 | YP_004134222 | Mesorhizobium ciceri biovar biserrulae WSM1271 | 1.574 ± 0.552 | 4.025 ± 1.449 | 2.6 | 5.60E-07 |
| TSV1 | ACM24772 | Sinorhizobium sp. NP1 | 2.568 ± 0.708 | 3.562 ± 2.925 | 1.4 | 0.0077 |
| TSV22 | YP_001314809 | Sinorhizobium medicae WSM419 | 0.417 ± 0.155 | 2.362 ± 1.445 | 5.7 | 3.24E-07 |
| TSV5 | YP_004134222 | Mesorhizobium ciceri biovar biserrulae WSM1271 | 1.066 ± 0.348 | 2.318 ± 1.210 | 2.2 | 0.0001 |
| TSV49 | ACF98123 | uncultured bacterium 1062 | 0.592 ± 0.292 | 2.222 ± 0.923 | 3.8 | 6.19E-09 |
| TSV11 | YP_004110634 | Rhodopseudomonas palustris | 0.984 ± 0.351 | 2.129 ± 0.688 | 2.2 | 1.91E-08 |
| TSV107 | ACF98152 | uncultured bacterium 1116 | 0.551 ± 0.256 | 1.889 ± 1.214 | 3.4 | 6.23E-07 |
| TSV66 | YP_473141 | Rhizobium etli | 0.393 ± 0.255 | 1.301 ± 0.889 | 3.3 | 1.60E-05 |
| TSV34 | NP_949481 | Rhodopseudomonas palustris | 0.535 ± 0.172 | 1.221 ± 0.862 | 2.3 | 0.0001 |
| TSV58 | NP_949481 | Rhodopseudomonas palustris | 0.562 ± 0.253 | 1.199 ± 0.789 | 2.1 | 0.0001 |
| TSV55 | YP_004134222 | Mesorhizobium ciceri biovar biserrulae WSM1271 | 0.472 ± 0.293 | 1.139 ± 1.197 | 2.4 | 0.0073 |
| TSV121 | ACF98123 | uncultured bacterium 1062 | 0.234 ± 0.133 | 0.966 ± 0.857 | 4.1 | 4.37E-06 |
| TSV123 | YP_004134222 | Mesorhizobium ciceri biovar biserrulae WSM1271 | 0.312 ± 0.217 | 0.906 ± 0.326 | 2.9 | 2.72E-06 |
| TSV43 | ACF98123 | uncultured bacterium 1062 | 0.310 ± 0.063 | 0.840 ± 0.465 | 2.7 | 2.45E-05 |
| TSV32 | ACF98152 | uncultured bacterium 1116 | 0.702 ± 0.170 | 0.795 ± 0.382 | 1.1 | 0.0076 |
| TSV24 | YP_473141 | Rhizobium etli | 0.428 ± 0.330 | 0.710 ± 0.310 | 1.7 | 2.79E-05 |
| TSV450 | NP_949481 | Rhodopseudomonas palustris | 0.157 ± 0.117 | 0.653 ± 0.650 | 4.1 | 0.0167 |
| TSV280 | ACF98123 | uncultured bacterium 1062 | 0.068 ± 0.050 | 0.623 ± 0.671 | 9.2 | 0.0003 |
| TSV137 | ACF98123 | uncultured bacterium 1062 | 0.194 ± 0.051 | 0.621 ± 0.419 | 3.2 | 0.0043 |
| TSV57 | YP_001314809 | Sinorhizobium medicae WSM419 | 0.112 ± 0.054 | 0.619 ± 0.349 | 5.5 | 7.80E-07 |
| TSV61 | NP_949481 | Rhodopseudomonas palustris | 0.289 ± 0.122 | 0.570 ± 0.385 | 2.0 | 2.11E-05 |
| TSV117 | YP_004134222 | Mesorhizobium ciceri biovar biserrulae WSM1271 | 0.207 ± 0.089 | 0.501 ± 0.291 | 2.4 | 3.89E-05 |
| TSV29 | ACF98123 | uncultured bacterium 1062 | 0.165 ± 0.110 | 0.474 ± 0.697 | 2.9 | 0.0213 |
| TSV178 | NP_949481 | Rhodopseudomonas palustris | 0.157 ± 0.059 | 0.409 ± 0.262 | 2.6 | 0.0052 |
| TSV175 | NP_949481 | Rhodopseudomonas palustris | 0.229 ± 0.050 | 0.388 ± 0.277 | 1.7 | 0.0023 |
| TSV220 | YP_004134222 | Mesorhizobium ciceri biovar biserrulae WSM1271 | 0.121 ± 0.037 | 0.387 ± 0.410 | 3.2 | 0.0004 |
| TSV288 | YP_001242619 | Bradyrhizobium sp. BTAi1 | 0.238 ± 0.261 | 0.387 ± 0.298 | 1.6 | 0.0209 |
| TSV25 | YP_004134222 | Mesorhizobium ciceri biovar biserrulae WSM1271 | 0.133 ± 0.054 | 0.349 ± 0.165 | 2.6 | 2.55E-05 |
| TSV179 | YP_004614534 | Mesorhizobium opportunistum | 0.073 ± 0.064 | 0.345 ± 0.364 | 4.7 | 0.0010 |
| TSV26 | YP_004134222 | Mesorhizobium ciceri biovar biserrulae WSM1271 | 0.206 ± 0.055 | 0.321 ± 0.204 | 1.6 | 0.0125 |
| TSV87 | ACF98152 | uncultured bacterium 1116 | 0.146 ± 0.074 | 0.318 ± 0.347 | 2.2 | 0.0103 |
| TSV248 | ACF98152 | uncultured bacterium 1116 | 0.090 ± 0.025 | 0.285 ± 0.132 | 3.2 | 2.08E-05 |
| TSV150 | NP_949481 | Rhodopseudomonas palustris | 0.121 ± 0.052 | 0.267 ± 0.097 | 2.2 | 1.13E-05 |
| TSV313 | ACF98152 | uncultured bacterium 1116 | 0.062 ± 0.053 | 0.244 ± 0.200 | 3.9 | 0.0056 |
| TSV131 | YP_004134222 | Mesorhizobium ciceri biovar biserrulae WSM1271 | 0.125 ± 0.048 | 0.239 ± 0.139 | 1.9 | 0.0025 |
| TSV269 | YP_004134222 | Mesorhizobium ciceri biovar biserrulae WSM1271 | 0.147 ± 0.049 | 0.235 ± 0.145 | 1.6 | 0.0016 |
| TSV200 | ACF98123 | uncultured bacterium 1062 | 0.060 ± 0.075 | 0.235 ± 0.181 | 3.9 | 0.0037 |
| TSV391 | ACF98123 | uncultured bacterium 1062 | 0.059 ± 0.035 | 0.225 ± 0.270 | 3.8 | 0.0134 |
| TSV323 | ACF98152 | uncultured bacterium 1116 | 0.096 ± 0.068 | 0.214 ± 0.112 | 2.2 | 0.0007 |
| TSV62 | YP_004134222 | Mesorhizobium ciceri biovar biserrulae WSM1271 | 0.080 ± 0.036 | 0.214 ± 0.218 | 2.7 | 0.0054 |
| TSV127 | Q01537 | Neorhizobium galegae | 0.073 ± 0.074 | 0.205 ± 0.217 | 2.8 | 0.0079 |
| TSV158 | YP_004134222 | Mesorhizobium ciceri biovar biserrulae WSM1271 | 0.122 ± 0.103 | 0.201 ± 0.117 | 1.6 | 0.0016 |
| TSV575 | ACF98123 | uncultured bacterium 1062 | 0.023 ± 0.022 | 0.200 ± 0.199 | 8.6 | 0.0204 |
| TSV580 | ACF98123 | uncultured bacterium 1062 | 0.042 ± 0.020 | 0.199 ± 0.164 | 4.7 | 0.0212 |
| TSV229 | NP_949481 | Rhodopseudomonas palustris | 0.113 ± 0.031 | 0.197 ± 0.124 | 1.7 | 0.0022 |
| TSV448 | YP_004134222 | Mesorhizobium ciceri biovar biserrulae WSM1271 | 0.023 ± 0.023 | 0.196 ± 0.185 | 8.6 | 0.0072 |
| TSV71 | YP_004134222 | Mesorhizobium ciceri biovar biserrulae WSM1271 | 0.039 ± 0.030 | 0.183 ± 0.132 | 4.7 | 0.0139 |
| TSV679 | ACF98123 | uncultured bacterium 1062 | 0.012 ± 0.016 | 0.179 ± 0.182 | 15.6 | 0.0027 |
| TSV533 | ACF98123 | uncultured bacterium 1062 | 0.040 ± 0.035 | 0.179 ± 0.119 | 4.5 | 0.0039 |
| TSV112 | ACF98088 | uncultured bacterium 1042 | 0.096 ± 0.034 | 0.176 ± 0.162 | 1.8 | 0.0042 |
| TSV818 | Q01537 | Neorhizobium galegae | 0.051 ± 0.044 | 0.175 ± 0.081 | 3.4 | 0.0018 |
| TSV430 | YP_004134222 | Mesorhizobium ciceri biovar biserrulae WSM1271 | 0.051 ± 0.035 | 0.171 ± 0.128 | 3.4 | 0.0270 |
| TSV156 | YP_004134222 | Mesorhizobium ciceri biovar biserrulae WSM1271 | 0.075 ± 0.059 | 0.156 ± 0.097 | 2.1 | 0.0056 |
| TSV170 | YP_004134222 | Mesorhizobium ciceri biovar biserrulae WSM1271 | 0.040 ± 0.030 | 0.152 ± 0.139 | 3.8 | 0.0237 |
| TSV464 | YP_004110634 | Rhodopseudomonas palustris | 0.058 ± 0.034 | 0.151 ± 0.137 | 2.6 | 0.0116 |
| TSV227 | ZP_04680002 | Ochrobactrum intermedium LMG 3301 | 0.086 ± 0.080 | 0.150 ± 0.106 | 1.7 | 0.0181 |
| TSV56 | YP_004134222 | Mesorhizobium ciceri biovar biserrulae WSM1271 | 0.065 ± 0.032 | 0.140 ± 0.079 | 2.1 | 0.0005 |
| TSV77 | YP_004134222 | Mesorhizobium ciceri biovar biserrulae WSM1271 | 0.068 ± 0.053 | 0.134 ± 0.076 | 2.0 | 0.0078 |
| TSV899 | YP_004134222 | Mesorhizobium ciceri biovar biserrulae WSM1271 | 0.044 ± 0.102 | 0.127 ± 0.187 | 2.9 | 0.0088 |
| TSV392 | YP_004134222 | Mesorhizobium ciceri biovar biserrulae WSM1271 | 0.032 ± 0.028 | 0.127 ± 0.062 | 4.0 | 0.0024 |
| TSV545 | NP_949481 | Rhodopseudomonas palustris | 0.061 ± 0.028 | 0.125 ± 0.089 | 2.0 | 0.0128 |
| TSV96 | YP_473141 | Rhizobium etli | 0.077 ± 0.047 | 0.121 ± 0.071 | 1.6 | 0.0085 |
| TSV241 | YP_004614534 | Mesorhizobium opportunistum | 0.054 ± 0.034 | 0.121 ± 0.108 | 2.2 | 0.0267 |
| TSV989 | ACF98123 | uncultured bacterium 1062 | 0.012 ± 0.015 | 0.106 ± 0.118 | 8.6 | 0.0075 |
| TSV330 | YP_004134222 | Mesorhizobium ciceri biovar biserrulae WSM1271 | 0.052 ± 0.041 | 0.105 ± 0.062 | 2.0 | 0.0073 |
| TSV538 | ZP_05742967 | Silicibacter sp. TrichCH4B | 0.021 ± 0.022 | 0.099 ± 0.104 | 4.7 | 0.0241 |
| TSV558 | ACF98123 | uncultured bacterium 1062 | 0.025 ± 0.014 | 0.093 ± 0.068 | 3.7 | 0.0234 |
| TSV823 | NP_949481 | Rhodopseudomonas palustris | 0.008 ± 0.011 | 0.087 ± 0.079 | 11.2 | 0.0010 |
| TSV416 | YP_004134222 | Mesorhizobium ciceri biovar biserrulae WSM1271 | 0.050 ± 0.073 | 0.072 ± 0.038 | 1.4 | 0.0129 |
| TSV296 | NP_949481 | Rhodopseudomonas palustris | 0.024 ± 0.018 | 0.067 ± 0.047 | 2.8 | 0.0100 |
| TSV869 | YP_004134222 | Mesorhizobium ciceri biovar biserrulae WSM1271 | 0.032 ± 0.023 | 0.067 ± 0.038 | 2.1 | 0.0160 |
| TSV1126 | YP_004134222 | Mesorhizobium ciceri biovar biserrulae WSM1271 | 0.011 ± 0.022 | 0.061 ± 0.051 | 5.5 | 0.0109 |
| TSV522 | NP_949481 | Rhodopseudomonas palustris | 0.006 ± 0.008 | 0.050 ± 0.043 | 8.2 | 0.0199 |
| TSV888 | ACF98152 | uncultured bacterium 1116 | 0.007 ± 0.013 | 0.046 ± 0.038 | 6.8 | 0.0166 |
| TSV1933 | NP_949481 | Rhodopseudomonas palustris | 0.005 ± 0.008 | 0.042 ± 0.054 | 9.0 | 0.0234 |
| TSV491 | ZP_03523451 | Rhizobium etli GR56 | 0.026 ± 0.030 | 0.041 ± 0.012 | 1.6 | 0.0173 |
| TSV388 | YP_004134222 | Mesorhizobium ciceri biovar biserrulae WSM1271 | 0.001 ± 0.003 | 0.038 ± 0.044 | 36.8 | 0.0270 |

Relative abundances are indicated as percentages (average ± standard deviation). The *p*-values are from Welch’s t-tests from ALDEx2 with Benjamini-Hochberg correction. The IDs of the nearest matching sequences from the FunGene *nirK* reference database and their source organisms are included.
