# Supplementary material for: Annual replication is essential in evaluating the response of the soil microbiome to the genetic modification of maize in different biogeographical regions
Source: PLoS One. 2019 Dec 17;14(12):e0222737. doi: 10.1371/journal.pone.0222737 (PMC6917299; doi:10.1371/journal.pone.0222737)
Supplement: Attachment — Submitted filename: Response to the 2ndreviewerscomments.docx [file pone.0222737.s005.docx]

Braunschweig, November 26, 2019

Dear Editor and Reviewers,

We are grateful for the thorough analysis of our manuscript. All four modifications suggested by Reviewer 2 were accepted and implemented into the 2^nd^ revision.

With kind regards,

Christoph C. Tebbe
